# Three-Dimensional Mass Transfer Modeling of Hydroquinone Adsorption on *Phragmites australis* Biochar

**DOI:** 10.3390/toxics11070639

**Published:** 2023-07-23

**Authors:** Shengli Shi, Aiguo Luo, Jianwei Hao, Shulian Xie, Jia Feng

**Affiliations:** 1 Department of Biological Science and Technology, Jinzhong University, Jinzhong 030600, China; shishengli@jzxy.edu.cn (S.S.); lag@jzxy.edu.cn (A.L.); haojianwei@jzxy.edu.cn (J.H.); 2 Shanxi Key Laboratory for Research and Development of Regional Plants, School of Life Science, Shanxi University, Taiyuan 030006, China; xiesl@sxu.edu.cn

**Keywords:** biochar, hydroquinone, adsorption kinetic, diffusional mass transfer model, 3D model

## Abstract

In this work, the overall adsorption kinetic process of hydroquinone on *Phragmites australis* biochar (PAC) was analyzed in depth. A 3D mass transfer model of pore volume and surface diffusion was established, and the diffusion mechanism was analyzed. The characterization results show PAC has a higher porosity value, which is conducive to the adsorption of hydroquinone. The adsorption process modeling results show that the combined effect of pore volume diffusion and surface diffusion promotes the total diffusion process of hydroquinone in the PAC particles, and the two mechanisms of pore volume and surface diffusion exist simultaneously. Under the different operating concentrations, the range of surface diffusion coefficient D_s_ is 2.5 × 10^−10^–1.74 × 10^−9^ cm^2^/s, and the contribution rate of surface diffusion SDCP% is close to 100%, which is much larger than pore volume diffusion, revealing that regardless of the contact time and position, surface diffusion occupies the main position in intraparticle diffusion.

## 1. Introduction

The Water pollution caused by organic pollutants has received widespread attention, especially that caused by phenolic compounds and their derivatives. Phenolic compounds and their derivatives are difficult to degrade, have a wide distribution, are carcinogenic to organisms, and can have toxic effects on ecosystems [1]. Hydroquinone, a phenolic derivative in which the two opposite positions of the benzene ring are occupied by hydroxyl groups, has been used in a variety of industries, including textiles, steel, petroleum refining, rubber epoxy resins and adhesives, and plastics [2]. Currently, there is evidence that hydroquinone has toxic effects on the eyes, skin, and respiratory tract.

Carbon adsorption is considered to be one of the most effective methods for the removal of organic pollutants from water. Biochar is prepared from different biomass wastes, making it a hot topic for adsorbent research due to its low cost, high adsorption efficiency, and simple preparation process [3,4]. However, at the same time, special attention should be paid to three aspects when designing biochar adsorption systems: the adsorption equilibrium isotherm and its dependence on the operating conditions (solution pH, temperature, and ionic strength), the total adsorption rate and mass transport mechanisms, and the interpretation and modelling of breakthrough curves [5]. Of these, kinetic studies are important for the interpretation and elucidation of adsorption phenomena as well as for the optimisation of adsorption system design. Kinetic models are usually divided into two categories, namely, adsorption reaction models and adsorption diffusion models.

The first considers that the adsorption kinetics is controlled entirely by the rate of solute adsorption on the adsorbent surface, which can be expressed in terms of chemical reaction rates. The models developed mainly include pseudo primary and pseudo secondary kinetic models, which are based on the assumption that the rate-limiting step in the overall adsorption process is the rate of adsorption on the adsorbent surface, while intraparticle diffusion and external mass transfer are neglected and no mass transfer parameters can be obtained, nor control steps for determining the adsorbate [6,7,8,9]. Therefore, we would like to use more realistic mathematical models to determine the mechanism of adsorbed mass transfer and to predict the diffusion process of adsorbed masses. Currently, the most realistic and reasonable approach to the study of adsorption kinetic processes is to use the second approach, the mass transfer diffusion model based on the adsorption mass transfer process [7,10,11], which considers diffusion through the liquid surrounding the adsorbent particles, diffusion in the liquid present in the pores and pore walls, and adsorption and desorption between the adsorbate and the active site [12,13,14]. The mass transfer diffusion model includes external mass transfer processes (external diffusion), intraparticle diffusion, and active site adsorption. Of these, intraparticle diffusion can, in turn, be controlled by pore volume diffusion (pore volume diffusion model, PVDM) and surface diffusion (surface diffusion model, SDM) or a combination of both mechanisms (pore volume and surface diffusion model, PVSDM 3D) [15,16].

However, despite the importance of mass transfer processes for a complete explanation of adsorption kinetics, a significant proportion of current studies have been developed based on adsorption reaction models, mainly due to the overly complex mathematical calculations of mass transfer diffusion models [14,17,18]. In this study, the kinetics of hydroquinone in *P. australis* biochar system will be simulated by simplifying the mass transfer diffusion model, using finite difference approximation methods to build PVSDM, SDM, and PVDM 3D models for comparison, to determine the detailed numerical solution of the PVSDM 3D model and the diffusion coefficient of hydroquinone. At the same time, the mass transfer of hydroquinone in the *P. australis* biochar system is explained in detail.

## 2. Materials and Methods

### 2.1. Materials

*P. australis* biochar (PAC) and other chemical reagents such as hydroquinone (molecular weight: 110.11 g/mol, λ = 289 nm) were of analytical purity.

### 2.2. Preparation

PAC was prepared by the microwave irradiation method previously reported in the laboratory [2]. Specifically, the collected *P. australis* were cleaned, cut, dried, and ground and then impregnated for 24 h using a solution of phosphoric acid (50%), followed by microwave irradiation in an experimental microwave oven for a certain period of time to carbonize the *P. australis* biochar samples of less than 75 µm in diameter, which were washed, dried, ground, and sieved to obtain a reserve of *P. australis* biochar.

### 2.3. Characterization

Characterisation analysis of PAC including scanning electron microscopy (SEM, S–4800, Hitachi, Tokyo, Japan), pore size analysis by BET (ASAP2460, Micromeritics, Norcross, GA, USA), X-ray diffraction (XRD, D2 Phaser, Bruker AXS, Karlsruhe, Germany), X-ray energy spectroscopy (XPS, Nexsa, Thermo Fisher Scientific, Waltham, MA, USA), and infrared spectroscopy (FTIR, Nicolet iS50, Thermo Fisher Scientific, Waltham, MA, USA) has been reported in a previous study by our group [2]. The true density of PAC was determined using a fully automated true density analyzer (AccuPyc II 1340, Norcross, GA, USA) with helium as the replacement medium using gas densitometry. The solid density obtained can be used to calculate the particle density and void fraction of the PAC using Equations (1) and (2) [7]:

(1) $$V_{p}=\frac{1}{\rho_{p}}-\frac{1}{\rho_{s}}$$

(2) $$\varepsilon_{p}=1-\frac{\rho_{p}}{\rho_{s}},$$

where *V_p_* (cm^3^/g) denotes the pore volume of PAC; *ρ_p_* (kg/m^3^) denotes the particle density of PAC; *ρ_s_* (kg/m^3^) denotes the solid density of PAC; and *ε_p_* denotes the void ratio of PAC.

### 2.4. Adsorption Experiment

Adsorption experiments were carried out using hydroquinone as the adsorbate. A total of 0.10 g of PAC was weighed into a 150 mL iodine measuring flask, and 100 mL of hydroquinone solution diluted to different concentrations (25, 50, and 100 mg/L) was added; the pH of the solution was set to the initial pH (pH = 6.83); the oscillation speed was set to 150 rpm; the temperature was set to 20, 30, and 40 °C in a thermostatic shaker; and samples were taken at different time points (10, 20, 40, 60, 90, 120, 150, 180, 240, 300, and 360 min) to determine the concentration of hydroquinone in the iodine measuring flask [4]. Specifically, a syringe was used to draw a certain amount of sample through a 0.45 μm cellulose acetate filter tip, and the absorbance was measured at 289 nm in a UV–Vis spectrophotometer (TU-1810, Pu-Analysis, Mudu Town, China) and the data analyzed. Three parallel test groups were set up for each experiment to minimize errors.

The adsorption rate *R* (%) of hydroquinone, the adsorption capacity *q_t_* of PAC at arbitrary time *t* and equilibrium time, and *q_e_* (mg/g) were calculated as shown in Equations (3)–(5):

(3) $$R(\%)=\frac{{(C}_{A0}-C_{e})}{C_{A0}}\times 100\%$$

(4) $$q_{t}=\frac{(C_{A0}-C_{A})V}{m}$$

(5) $$q_{e}=\frac{{(C}_{A0}-C_{e})V}{m},$$

where *R* (%) represents the adsorption rate of hydroquinone; *C_A_*_0_ and *C_A_* (mg/L) represent the initial concentration of hydroquinone and the concentration at any time *t*, respectively; *C_e_* (mg/L) represents the concentration of hydroquinone at adsorption equilibrium; *q_t_* (mg/g) represents the amount of *P. australis* biomass material adsorbed at any time *t*; *q_e_* (mg/g) represents the amount of *P. australis* biomass material adsorbed at adsorption equilibrium; *V* (mL) represents the volume of hydroquinone; and *m* (g) represents the mass of the *P. australis* biomass material.

## 3. Mathematical Modeling

### 3.1. Isotherm Models

Adsorption isotherm equilibrium is the curve of the relationship established between adsorbate concentration and adsorbent when adsorption reaches saturation at a defined temperature condition [19]. In experiments, the discussion of the adsorption mechanism and the investigation of the relationship between adsorbate and adsorbent mass can be obtained by the analysis of isotherms obtained at different temperatures. The Langmuir isotherm model assumes that there are uniformly distributed adsorption sites with equal energy on the adsorbent surface, that the adsorbate molecules are independent of each other, and that adsorption ceases when a monolayer on the adsorbent surface reaches saturation [19,20,21,22], and it is widely used to reflect a homogeneous monolayer adsorbent surface. The Freundlich isotherm model is an empirical model that assumes an uneven energy distribution on the adsorbent surface and that adsorbate molecules can attract or repel each other, forming a multilayer adsorption. It is suitable for non–uniform systems with a non–uniform heat distribution of adsorption [19,22,23,24,25,26]. Two isotherm model equations are shown in (6) and (7):

(6) $$q_{e}=\frac{q_{m}k_{L}c_{e}}{1+k_{L}c_{e}}$$

(7) $$q_{e}={k_{F}c_{e}}^{1/n},$$

where *q_m_* (mg/g) indicates the maximum adsorption of the hydroquinone by the *P. australis* biomass material as calculated by the Langmuir isotherm model; *k_L_* (L/mg) indicates the adsorption constant; *k_F_* (mg/g (L/mg) 1/n) indicates the adsorption constant; and 1/n indicates the strength of the adsorption effect and the evaluation of the heterogeneity of the adsorbent when n < 1, indicating favourable in the range of adsorption concentrations, and when n > 1, indicating favourable only in the high concentration range [27].

### 3.2. 3D Mass Transfer Model

In practical adsorption processes, although the multiple parameters involved in the kinetics of adsorption are very complex, the mass transfer process, starting with complete agitation, can be approximated to the following three basic steps. The first step is external diffusion, where the adsorbate transfer is through the liquid membrane surrounding the adsorbent and is therefore also referred to as membrane diffusion; the second step is internal diffusion, and this step describes the entry of the adsorbate into the internal surface of the adsorbent pores after passing through external diffusion and is also referred to as intraparticle diffusion; and the third step represents the binding process of the adsorbate at the active site on the adsorbent surface [28]. The most common mathematical models are the primary and secondary kinetic models, which are applied by considering only the third stage and ignoring the two initial stages of the mass transfer process.

The sorbent mass transfer diffusion model is based on the following factors: (1) both pore volume and surface diffusion are the main drivers of sorbent diffusion within the sorbent particles; (2) the rate of sorption of sorbent at the active site is instantaneous; (3) the sorbent material particles are rigid, homogeneous, and isotropic; and (4) in contrast to diffusion, the internal convective transport generated by the adsorbent mass is negligible [28]. Based on the above assumptions, a set of model equations for pore volume and surface diffusion during mass transfer, as well as initial and boundary conditions, were obtained, as shown in Equations (8)–(11) [28,29]:

(8) $$\frac{dC_{A}}{dt}=-\frac{mSk_{L}}{V}(C_{A}-C_{Ap\left|r=R\right.})$$

(9) $$\varepsilon_{p}\frac{\partial C_{Ap}}{\partial t}+\rho_{p}\frac{\partial q}{\partial t}=\nabla \cdot (D_{ep}\nabla C_{Ap}+D_{s}\rho_{p}\nabla q)$$

(10) $$t=0,C_{A}=0,C_{Ap}=0$$

(11) $$D_{s}\rho_{p}\nabla q+D_{ep}\nabla C_{Ap\left|r=R\right.}=k_{L}(C_{A}-C_{Ap\left|r=R\right.}),$$

where Equations (8)–(11) denote the set of equations for the three-dimensional pore volume and surface diffusion models; *t* (s) denotes time; *r* (cm) denotes the depth of the adsorbate into the interior of the PAC; *R* (cm) denotes the PAC particle size; *C_A_* and *C_Ap_* (mg/L) denote the concentration of the adsorbate in solution and inside the PAC, respectively; *q* (mg/g) denotes the adsorption volume of PAC; *k_L_* (cm/s) denotes the external mass transfer coefficient; *D_ep_* and *D_s_* denote the diffusion coefficients for pore volume diffusion and surface diffusion, respectively; and *C_Ap_*_|*r=R*_ denotes the solute concentration within the particle calculated at the boundary; in addition, assuming that the adsorption rate of the adsorbate at the active site is instantaneous, the relationship between *q* and *C_AP_* can be evaluated by the adsorption isotherm model.

In order to solve the 3D pore volume and surface diffusion model equations set (8)–(11), the values of the parameters in the mass transfer process, i.e., *k_L_*, *D_s_* and *D_ep_*, must be obtained. Where the *k_L_* values can be obtained by using the theory proposed by Furusawa and Smith [29,30] in 1973, as shown in Equation (12):

(12) $$k_{L}=-\frac{V}{mS}{\left(\frac{d\theta }{dt}\right)}_{t\to 0},$$

where *θ* = *C_A_*/*C_A_*_0_; *S* (cm^2^/g) denotes the specific surface area per unit mass of adsorbent; and the right half of Equation (12), in parentheses, indicates the slope of the concentration decay at *t* = 0, which can be estimated from the slopes at the two time points *t* = 0 and *t* = 5.

The value of *D_ep_* can be calculated from Equation (13) [29]:

(13) $$D_{ep}=\frac{D_{AB}\varepsilon_{p}}{\tau },$$

where *τ* denotes the curvature factor of the adsorbent material and based on the results of Leyva-Ramos and Geankoplis [16]; 3.5 is recommended as the curvature factor for PAC; *D_AB_* denotes the diffusivity of the adsorbent molecules in aqueous solution and can be calculated from Equation (14) [7,31]:

(14) $$D_{AB}=7.4\times 10^{-8}\left[\frac{{\left(\varphi M_{B}\right)}^{0.5}T}{\eta_{B}V_{A}^{0.6}}\right],$$

where *φ* and *M_B_* (g/mol) denote the water association parameter of 2.60 and the molar mass of 18.02, respectively; *T* denotes the ambient temperature of 303.15 K; the viscosity of water *η_B_* = 0.904 cp; and *V_A_* (cm^3^/mol) denotes the molar volume of the adsorbate, which can be calculated by the Le Bas method [32].

The value of *D_s_* was estimated from the diffusion model data with experimental data by the least squares optimization method and was calculated as shown in Equation (15) [29]:

(15) $$error={\int_{t=0}^{t=300}\left(C_{Aexp}-C_{Acal}\right)}^{2}dt,$$

where *C_Aexp_* and *C_Acal_* (mg/L) denote the concentration of the sorbent obtained by the diffusion model and experimentally calculated at different times, respectively. The mass transfer process of the sorbent inside the PAC was simulated by using the finite element method, COMSOL Multiphysics 5.4 and other mapping software.

## 4. Results and Discussion

### 4.1. Characterisation of Solid Density

The solid and particle densities and void fraction results for PAC are shown in Table 1. The solid density of PAC is 1.63 kg/m^3^, the particle density is 0.52 kg/m^3^, and the void fraction is 0.68. From the results, it can be seen that PAC has a large internal pore space, which is conducive to the adsorption process.

### 4.2. Adsorption Isotherm

**Figure 1:**
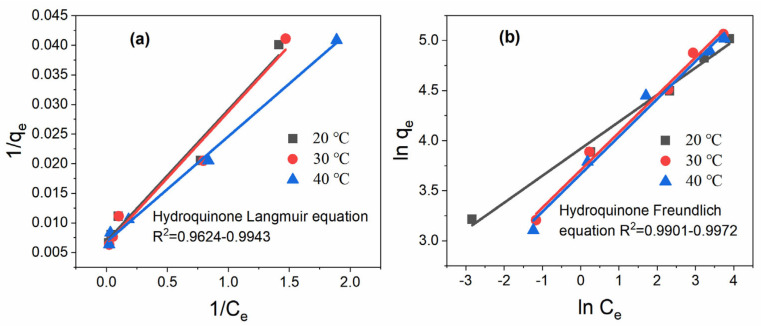
(**a**) Langmuir; (**b**) Freundlich adsorption isotherm models of hydroquinone on PAC.

Figure 1a,b and Table 2 show the results of the fitting of two different isotherm models, Langmuir and Freundlich, to simulate the adsorption of hydroquinone on the surface of PAC. From the results, it can be seen that the isotherm data for the adsorption of hydroquinone on PAC can be fitted well using both the Langmuir and Freundlich isotherm models, with the Freundlich isotherm model providing a better fit. The analysis of the relevant parameters in Table 2 also shows that the Adj R^2^ value of the Freundlich isotherm model (0.9901–0.9972) is higher than that of the Langmuir model, and the n value greater than 1 is the dominant adsorption. The results indicate that the Freundlich isotherm model can better describe the adsorption behavior of hydroquinone on PAC, which has a heterogeneous surface, and the adsorption of hydroquinone on the surface of PAC is heterogeneous and belongs to the adsorption process of the multi-molecular layer. In addition, the calculated results from the Langmuir isotherm model show that the maximum adsorption capacity of hydroquinone on PAC is 158.73 mg/g for a single layer, which possesses a strong adsorption capacity.

### 4.3. Simulation of Intraparticle Adsorption and Mass Transfer Processes

In order to further investigate the mass transfer process of hydroquinone inside PAC and to understand the key steps in controlling diffusion, the mass transfer process of hydroquinone inside PAC was simulated and analyzed using the pore volume (PVDM) and surface diffusion model (SDM), as well as the pore volume diffusion model in combination the surface diffusion model (PVSDM).

#### 4.3.1. Concentration Decay Curve for Hydroquinone

**Figure 2:**
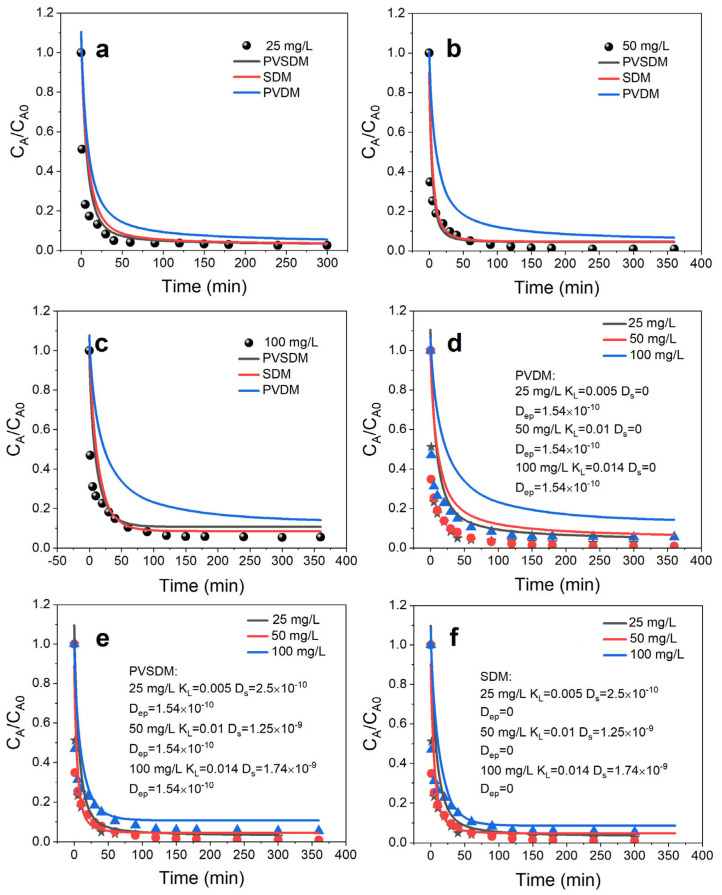
Concentration decay curves of hydroquinone on PAC: (**a**) 25 mg/L; (**b**) 50 mg/L; and (**c**) 100 mg/L hydroquinone fitted by pore volume and surface diffusion model, surface diffusion model, and pore volume diffusion model; 25, 50, and 100 mg/L hydroquinone fitted by (**d**) pore volume and surface diffusion model, (**e**) pore volume diffusion model, and (**f**) surface diffusion model.

The time taken for the sorbent to reach equilibrium is critical to the design of the adsorption system and is highly dependent on the concentration of the sorbent, temperature, and other conditions of system operation. Figure 2 shows the concentration decay curve for different concentrations of hydroquinone with increasing time; the concentration decreases with increasing time and the equilibrium adsorption amounts are 24.35, 49.56, and 94.55 mg/g, respectively, when the adsorption time reaches 180 min and the hydroquinone reaches equilibrium in the adsorption system.

#### 4.3.2. Concentration Decay Curve for Hydroquinone

First, the concentration decay curves under experimental conditions with different initial concentrations of hydroquinone were simulated using a pore volume diffusion model, which assumes negligible surface diffusion, D_s_ = 0. According to the set of model equations (Equations (8)–(11)), the values of the external mass transfer coefficient k_L_ and the effective pore volume diffusion coefficient D_ep_ need to be obtained, as shown by Equations (12) and (13), respectively. The values of k_L_ were calculated to obtain a range of 0.5 × 10^−2^ to 1.4 × 10^−2^ cm/s, and the estimated value of D_ep_ was 1.54 × 10^−10^ cm^2^/s. Figure 2a–d represents the values of k_L_ using pore-volume-diffusion-model-predicted and experimentally obtained concentration decay curves. It can be seen from the results that the use of the pore volume dispersion model does not adequately explain the experimental data obtained; the pore volume dispersion model overestimates the experimental data, and the difference becomes more pronounced as the initial concentration increases.

#### 4.3.3. Simulation Applications of Pore Volume and Surface Diffusion Models

The concentration decay curves for different initial concentrations of hydroquinone under experimental conditions were simulated using a pore volume and surface diffusion model. It assumes that intraparticle diffusion is caused by both pore volume and surface diffusion mechanisms. In addition, the model assumes that the diffusion of hydroquinone within the PAC particles is driven by both the concentration gradient of the fluid phase within the pore space and the mass absorption gradient of the solid phase. Similarly, in addition to the two mass transfer parameters k_L_ and D_ep_, values for D_s_ are also obtained in the pore volume and surface diffusion models. By analyzing the data fitted to the model with the experimental data, the values of D_s_ obtained using Equation (15) range from 2.5 × 10^−10^ to 1.74 × 10^−9^ cm^2^/s. As shown in Figure 2a–c,e, the values of D_s_ obtained using the pore volume and concentration decay curves of hydroquinone within the PAC adsorption system were obtained by fitting the surface diffusion model. As shown in Figure 2e, the experimental data for the adsorption of hydroquinone on PAC can be well fitted using the pore volume and surface diffusion models.

**Figure 3:**
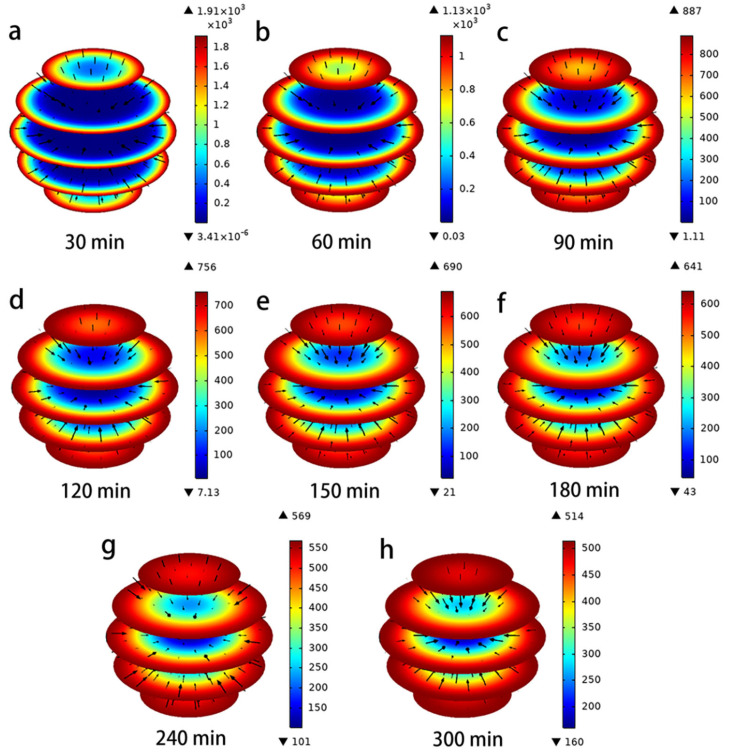
(**a**–**h**) Evolution of hydroquinone in PAC intraparticle profiles.

In addition, Figure 3 represents the three-dimensional evolution of the adsorption process of hydroquinone on the PAC surface predicted via the pore volume and surface diffusion models for an initial concentration of 25 mg/L (arrows indicate direction; colors indicate size). It can be clearly seen that in the initial stage of adsorption, the hydroquinone molecules mainly stay on the surface of the activated carbon and the direction of mass transfer converges towards the interior of the PAC particles. As the adsorption time increases, the hydroquinone molecules continue to diffuse into the interior of the PAC, eventually reaching an equilibrium state.

In order to confirm as well as distinguish the diffusion mechanism of hydroquinone within PAC, the dominant factor in the diffusion mechanism can be determined by calculating the mass transport contribution caused by the pore volume *N_AP_* and the corresponding surface diffusion *N_AS_*. The estimating equations shown in Equations (16) and (17) [28]:

(16) $$N_{AP}=-D_{ep}\nabla C_{Ap}.$$

(17) $$N_{AS}=-D_{s}\rho_{p}\nabla q$$

**Figure 4:**
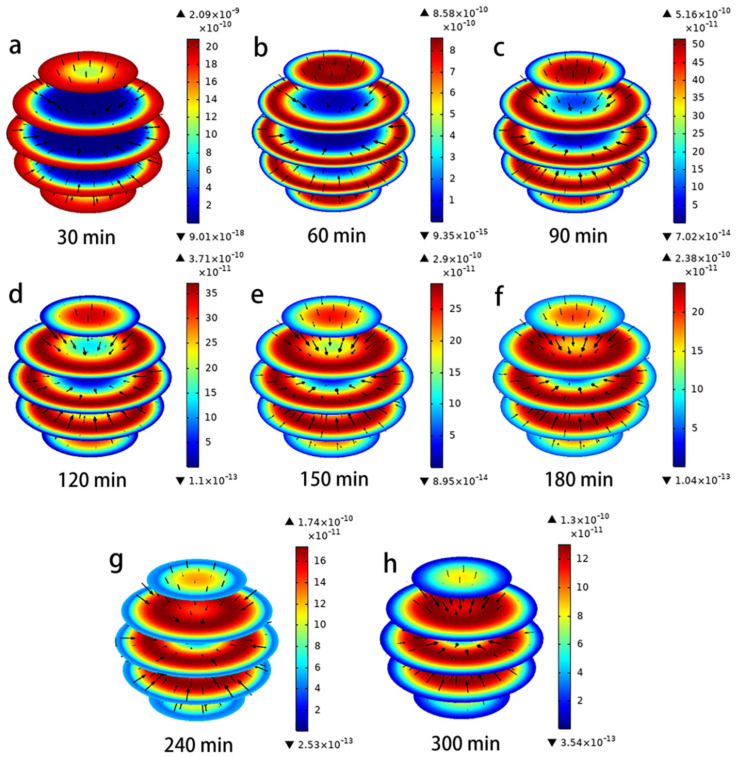
(**a**–**h**) Evolution of magnitude and direction of N_AP_ during the adsorption of hydroquinone in PAC.

**Figure 5:**
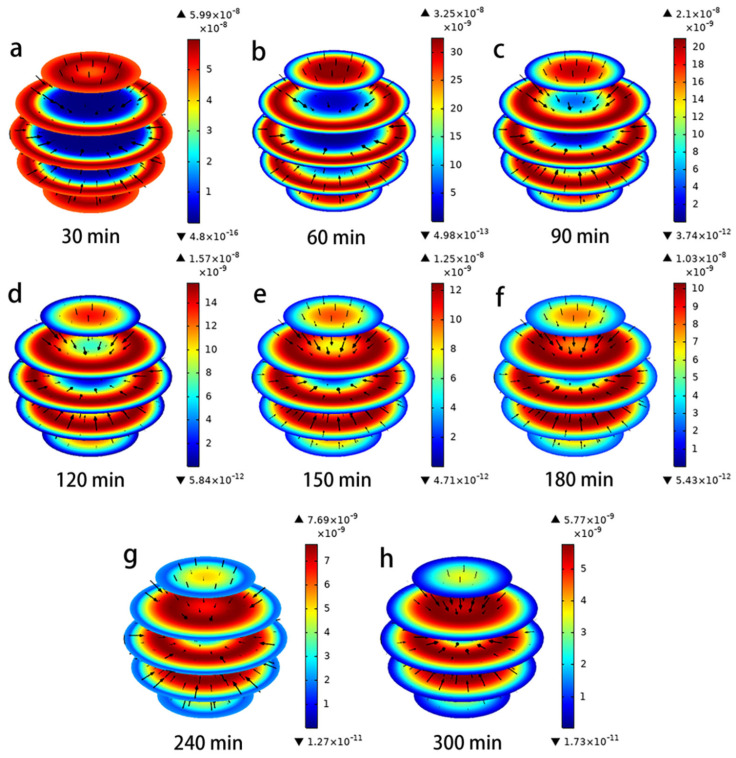
(**a**–**h**) Evolution of magnitude and direction of N_AS_ during the adsorption of hydroquinone in PAC.

The evolution of the size and direction of the pore volume *N_AP_* and surface diffusion *N_AS_* under different time conditions is represented in Figure 4 and Figure 5. The direction of the flux (black arrows) converges towards the interior of the PAC particles, indicating that the lowest concentration of hydroquinone can be reached in the interior. In the Figures, the red color represents the maximum values of *N_AP_* and *N_AS_*, and the blue color represents the minimum. It is clear from these data that both mass transport mechanisms occur simultaneously, but their magnitudes are a function of time and position within the PAC particles, which is consistent with Frhlich’s findings [15,28]. The maximum values of N_AP_ and N_AS_ are obtained near the outer surface of the PAC particle during the initial time period; thereafter, they continue to increase near the center of the particle over a long time. Furthermore, the difference between the magnitudes of N_AP_ and N_AS_ can be seen from Figure 4 and Figure 5, when the time is 60 min, the maximum N_AP_ is 8.58 × 10^−10^ mg/cm^2^/min, and the maximum N_AS_ is 3.25 × 10^−8^ mg/cm^2^/min, the N_AS_ value is two orders of magnitude higher than the *N_AP_* value. This indicates that surface diffusion flux is more important than pore volume diffusion [29]. From the characterization results of PAC (SEM, FTIR, XPS, XRD), it was found that the PAC surface is rich in adsorption active sites, which may be the reason for the surface diffusion flux being larger than the pore volume diffusion [2,5].

It is essential to assess the contribution of each diffusion mechanism to the overall intraparticle diffusion and the percentage contribution of surface diffusion (*SDCP*%) to the total intraparticle diffusion. It can be calculated based on the contribution of *N_AP_* and *N_AS_* to the intraparticle diffusion drive using the following equation (Equation (18)) [28]:

(18) $$SDCP\%=\frac{\left\|N_{AS}\right\|}{\left\|N_{AS}\right\|+\left\|N_{AP}\right\|}\times 100$$

(19) PDCP%=1−SDCP%.

**Figure 6:**
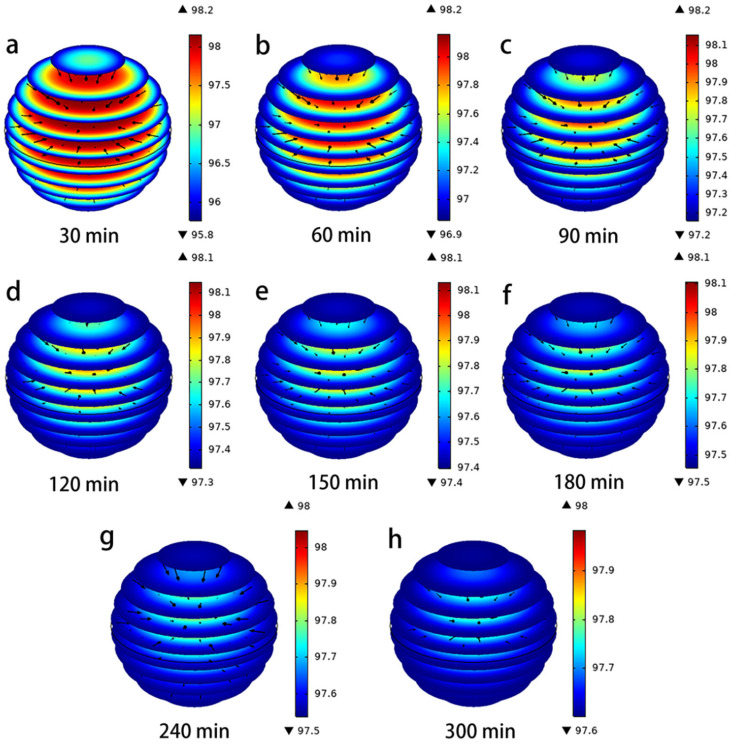
(**a**–**h**) Evolution of SDCP% for hydroquinone transport inside PAC as a function of contact time.

As can be seen from the variation of *SDCP*% with contact time in Figure 6, the *SDCP*% values consistently remained above 95% and close to 100%, regardless of time and radial position. Moreover, as can be seen from the variation of *SDCP*% values in the graph, the size of the *SDCP*% increased from 95.80 to 98.20% over the 30–360 min range. When the adsorption time increased, the maximum value of *SDCP*% gradually shifted towards the interior of the PAC particles, while the importance of the pore volume diffusion *PDCP*% became closer to the surface. The results indicate that pore volume diffusion and surface diffusion act together to drive the total diffusion of hydroquinone within the PAC particles. The contribution of surface diffusion is close to 100% regardless of the contact time and location, thus confirming that surface diffusion is the main mechanism of intraparticle diffusion. This phenomenon may be due to the fact that during the initial stage of adsorption, the adsorbate molecules first diffuse quickly towards the center of the particle via surface diffusion for a short time, and then diffuse via pore volume diffusion and thus towards the effective adsorption site once the adsorption site is occupied and closer to equilibrium [5]. In addition, the abundant adsorption sites on the PAC surface facilitate the surface diffusion [2,15]. Thus, at higher adsorption volumes and closer to equilibrium, the adsorbate diffuses mainly through the pore volume, which also indicates that the importance of surface diffusion gradually shifts towards the interior of the PAC particles, while the importance of pore volume diffusion becomes closer to the surface [29].

In addition, in terms of the contribution of surface diffusion, there is a tendency for surface diffusion to increase overall as time increases, which could explain this behavior from the start by adsorbing molecules to sites that require higher energy and where these adsorbed molecules do not have sufficient energy to desorb from one site and diffuse to another adsorption site [29]. Once the active sites with higher adsorption energies are occupied, molecules will be adsorbed on sites with lower adsorption energies; therefore, more molecules can be desorbed and transferred from one site to another [5].

## 5. Conclusions

In this study, a three-dimensional adsorption mass transfer model (PVSDM 3D) of hydroquinone on biochar (PAC) was developed. The PVSDM 3D model was able to better predict the concentration decay curve in the adsorption system. The adsorption of hydroquinone on PAC is a multi–molecular layer adsorption process which is caused by a combination of interactions between phenolic contaminant molecules and PAC surface. In the simulated adsorption process using a three-dimensional mass transfer model, the value of the surface diffusion coefficient increased from 2.5 × 10^−10^ to 1.74 × 10^−9^ cm^2^/s, verifying that a faster adsorption rate will be generated as the concentration increases. Moreover, the contribution of surface diffusion was close to 100% regardless of the contact time and location, confirming the dominance of surface diffusion in intraparticle diffusion in this study. The development of a three-dimensional mass transfer model will help to more accurately model and explain the adsorption and mass transfer of adsorbate within the adsorbent.

## Figures and Tables

**Table 1:** Solid density and other parameters of PAC.

Project	Value
*ρ_s_* (kg/m^3^)	1.63
*ρ_p_* (kg/m^3^)	0.52
*ε_p_*	0.68

**Table 2:** Related parameters of adsorption isotherm models at 20, 30 and 40 °C of hydroquinone on PAC.

Isotherm Models		Hydroquinone		
		20 °C	30 °C	40 °C
Langmuir	q_m_ (mg/g)	147.06	156.25	158.73
	k_L_ (L/mg)	0.3063	0.3170	0.3535
	Adj R^2^	0.9647	0.9624	0.9943
Freundlich	k_F_ (mg/g(L/mg)^1/n^)	50.32	40.65	39.05
	n	3.71	2.68	2.67
	Adj R^2^	0.9972	0.9969	0.9901

## Data Availability

Data are contained within the article.
